# The conditions of failing and successful plant invasions with free boundary

**DOI:** 10.1038/s41598-025-02695-4

**Published:** 2025-05-23

**Authors:** Rong Li, Linling Zhu, You Zhou

**Affiliations:** School of Mathematical Science, Yangzhou University, Yangzhou, 225002 China

**Keywords:** Reaction-diffusion model, Free boundary, Plant invasions, Spreading and vanishing, Forest ecology, Invasive species, Population dynamics

## Abstract

In this paper, a reaction-diffusion model is proposed to investigate the growing process of invasive plants. Two free boundaries are introduced to describe the spreading frontiers are caused only by the seeds. The main objective is to realize the variation of the invasive plants and the free boundaries. Similar to the basic reproduction number of epidemic diseases, we define the threshold parameters $J_0$ and $J_0^F(t)$ to discuss the dynamic behaviors of equilibrium solutions of invasive plants model with free boundary conditions. Based on them, we give the sufficient conditions for whether plants invade successfully or not. We show that the invasive plants will successfully persist in the new environment if $J^F_0(0)<1<J_0$ with large initial values or $J_0^F(t_0)\ge 1 ~(\exists t_0\ge 0)$, while failing and dying out in the long run if $J^F_0(0)<1$ with small initial values or $J_0\le 1$.

## Introduction

In environmental issues, the study of negative biological invasions lie in an important position because its great harm to the world on the aspect of social economy, local environment and so on. In China, plant invasion is quite serious. It is reported that there are more than 380 kinds of invasive plants at present, threatening agricultural ecosystem and the environment of habitats for raising livestock and fish, which even affects people’s health. The essential problems in the theoretical studies of this subject are to know the growing or decaying process of invasive plants and mathematical structures, so that they could be well-controlled according to the research results. In fact, many researchers have already attempted to solve these problems by means of various scientific methods^1–10^. Statistical methods, such as the direct gradient analysis and regression analysis^1^, maximum likelihood estimation^2,3^, were applied to study the the synecology and characteristics of tree species. The computer simulation was carried out to create a complete model of forest growth^4,5^. One of the most challenging aspects of employing statistical methods and conducting computer simulations is the acquisition of reliable and comprehensive data. Unfortunately, there are usually very limited empirical data, since in most cases any useful field data need to cover vast areas and long time spans, and hence are extremely difficult and expensive to obtain. In view of the non-experimental characteristic, mathematical modeling is one of the important tools to investigate the transmission laws of plant invasion and then to prevent and control. Some scholars utilized mathematics models to investigate kinetic process of the individual trees, trees in a plot of forest, and even of all the trees which interact one another in a forest^6–10^.

In this paper, we introduce the ASCS (age-structured continuous-space) plants kinematic model which was first proposed by Kuznetsov et al.^7^ to describe the dynamic growing process of invasive plants. In modeling, the plants are simply divided into two age classes, namely, young age and old age. The model is structured as follows:

1 $$\begin{aligned} \left\{ \begin{array}{lll} u_{t}=\delta \beta w-\gamma (v)u-fu, \ & \text{ in }\ \Omega \times (0,\infty ),\\ v_{t}=fu-hv, \ & \text{ in }\ \Omega \times (0,\infty ),\\ w_{t}=\alpha v-\beta w+p w_{xx}, \ & \text{ in }\ \Omega \times (0,\infty ),\\ w(x,t)=0, \ & \text{ on }\ \partial \Omega \times (0,\infty ),\\ u(x,0)=u_{0}(x),\ v(x,0)=v_{0}(x), \ w(x,0)=w_{0}(x), \ & \text{ in }\ \Omega . \end{array} \right. \end{aligned}$$

Here, $u(x,t)$ and $v(x,t)$ denote the plant densities of the young and old age classes in the domain $\Omega$ at time *t*, respectively. The other unknown function $w(x,t)$ represents the density of seeds in the air over $\Omega$. The first and second equations separately describe the growth of these two class plants, where $\delta$ is an establishment rate of seeds, satisfying $0<\delta \le 1$ ; $\gamma (v)>0$ is the mortality of young plants, which is allowed to depend on the old-plant density $v$; $f>0$ is an aging rate and $h>0$ is a mortality of old generation plants. Meanwhile, the third equation describes the kinetics of seeds, where $p>0$ is the seed-diffusion rate in the air and $\alpha>0, \beta >0$ are the seed production and seed deposition rates, respectively. Specially, for $w$, the homogeneous Dirichlet condition is imposed on $\partial \Omega$. The initial functions $u_0(x)$, $v_0(x)$ and $w_0(x)$ are given nonnegative functions in $\Omega$.

In the initial paper^7^, the authors discussed the stationary solutions and travelling wave solutions by reducing equations in (1) into a system of cross-diffusion type. After that, many further studies have been done. For instance^11^, Wu and Lin studied the exponential stability and instability of nonnegative steady-state solutions to the modified system of (1) with the production term $\alpha v$ being replaced by the spatial average $\alpha \int _\Omega v \text {d} x$. Since a span of time is needed when a seed comes up and grows into a “young” plant, Lin and Liu used the term of time delay $\delta \beta w(x, t-\tau ), \tau >0$ to modify the establishment term $\delta \beta w$^12^. They give the global existence and boundedness of solutions and analyze the equilibrium solutions. Besides, Chuan et al.^13^ and Shirai et al.^14^ were clear in the structure of three kinds of $\omega$-limit sets of trajectories. Considering how the mathematical structure of the dynamical system changes after incorporating the effect in the seed establishment process, Mola and Yagi were concerned with a forest kinematic model with memory^15^.

Without doubt, global solutions and travelling wave solutions are good ways to understand the mathematical structure of solutions in the fixed area. However, the plant may die out in some cases, for example, when the initial data or survival area is sufficiently small. In addition, the solution of system (1) is always nonnegative and nontrivial as long as $t>0$, regardless of the initial data of plant quantity or existent area, which contradicts the fact that the invasive species can not spread to the whole space immediately. Considering these aspects, Du and Lin^16^ proposed a different approach to the understanding of the spreading of species in 2010. They introduced free boundaries into the diffusive logistic model to describe the expanding fronts, and obtain a spreading-vanishing dichotomy for the model. More biological interpretations of free boundaries are referred to references^17,18^. Their work inspires a lot of researchers and the idea has been applied in many fields to describe the gradual expanding process and changing of domain. For example, the spatial transmission of epidemics of infectious diseases^19–24^, the information diffusion in online social networks^25,26^, the spreading of species in ecological models^27–32^.

Inspired by the former work, we introduce free boundary to the ASCS model with $\gamma (v)=\gamma v,$ where $\gamma$ is a sufficiently small positive constant for simplicity. It indicates the strong viability of the invasive plants. Now, the plant invasions model with double free boundaries in one-dimension space is in the following form:

2 $$\begin{aligned} \left\{ \begin{array}{lll} u_{t}=\delta \beta w-\gamma vu-fu, \ & s_l(t)<x<s_r(t), \ t>0,\\ v_{t}=fu-hv,\ & s_l(t)<x<s_r(t), \ t>0,\\ w_{t}=\alpha v-\beta w+p w_{xx},\ & s_l(t)<x<s_r(t), \ t>0,\\ u(x,t)=v(x,t)=w(x,t)=0, \ & x=s_l(t)\ \text{ or }\ s_r(t),\ t>0,\\ s_l(0)=-s_0, \ s_l'(t)=-\mu w_x(s_l(t), t), \ & t>0, \\ s_r(0)=s_0, \ s_r'(t)=-\mu w_x(s_r(t), t), & t>0,\\ u(x,0)=u_{0}(x),\ v(x,0)=v_{0}(x), \ w(x,0)=w_{0}(x), & -s_0\le x\le s_0, \end{array} \right. \end{aligned}$$

where $s_l(t)$ and $s_r(t)$ are moving left and right boundary, respectively, which will be determined together with the solution. $s_0$ and $\mu$ are both positive constants. $-s_0,s_0$ are left and right initial boundary, respectively. $\mu$ donates the spreading ability of the moving boundaries. $s_l'(t)=-\mu w_x(s_l(t), t)$ and $s_r'(t)=-\mu w_x(s_r(t), t)$ are the conditions on the left and right fronts, respectively, which imply that $s_l(t)$ and $s_r(t)$ grow at a rate that is proportional to seed quantity gradient at the fronts^27^. The initial functions $u_0, v_0$ and $w_0$ are non-negative, non-trivial and satisfy

3 $$\begin{aligned} \left\{ \begin{array}{ll} u_0, v_0, w_0 \in C^{2}([-s_0, s_0]), u_0(\pm s_0)= v_0(\pm s_0) =w_0(\pm s_0)=0,\\ u_0, v_0, w_0>0 \ \text{ in } \ (-s_0, s_0). \end{array} \right. \end{aligned}$$

The remainder of this paper is organized as follows. In “Existence and uniqueness”, we first apply the contraction mapping theorem to prove the global existence and uniqueness of the solution to the problem (2). Then we prove several free boundary properties associated with our problem. In “Long time behavior of the solution”, the comparison principals will be present first, and then we turn to prove whether the plants fail to expand or invade successfully in a new environment in a long run. Finally, we give a brief discussion and some numerical simulations in “Numerical simulations and discussion”.

## Existence and uniqueness

In this section, we first prove local existence and uniqueness of the solution to problem (2) and then we give some suitable estimates about the solution and the double free boundary fronts, so that we could get further with the global existence of the solution. In the last part of this section, we display the property of the double free boundaries.

### Theorem 1

*For any given* $u_0, v_0$ *and* $w_0$ *satisfying* (3) *and any* $\alpha \in (0,1)$, *there is a positive number* *T* *such that problem* (2) *admits a unique solution*

$$(u,v,w;s_l,s_r)\in [C^{1+\alpha ,(1+\alpha )/2}(D_{T})]^3\times [C^{1+\alpha /2}([0,T])]^2,$$

*moreover*,

4 $$\begin{aligned} \Vert u\Vert _{C^{1+\alpha , (1+\alpha )/2}(D_{T})}+\Vert v\Vert _{C^{1+\alpha , (1+\alpha )/2}(D_{T})}+\Vert w\Vert _{C^{1+\alpha , (1+\alpha )/2}(D_{T})} \nonumber \\ +\Vert s_l\Vert _{C^{1+\alpha /2}([0,T])}+\Vert s_r\Vert _{C^{1+\alpha /2}([0,T])}\le K, \end{aligned}$$

*where* $D_{T}=\{(x,t)\in \mathbb {R}^2:x\in [s_l(t), s_r(t)],t\in [0,T]\}$, *K* *and* *T* *are positive constants depending on* $s_0$, $\alpha$, $\Vert u_0\Vert _{C^{2}([-s_0, s_0])}$, $\Vert v_0\Vert _{C^{2}([-s_0, s_0])}$ *and* $\Vert w_0\Vert _{C^{2}([-s_0, s_0])}$.

### Proof

First, we try to represent $u$ and $v$ with regard to $s_l, s_r$ and $w$ since the lack of diffusion terms in the first and second equations of (2). For any given $T>0$, we define

$$\mathscr {S}_{lT}=\{s_l\in C^1([0,T]): s_l(0)=-s_0, s_l'(t)\le 0, 0\le t\le T\}$$

and

$$\mathscr {S}_{rT}=\{s_r\in C^1([0,T]): s_r(0)=s_0, s_r'(t)\ge 0, 0\le t\le T\}.$$

Introduce the extension mapping $E_t$ by $E_t(\varphi )(x,t)=\varphi (x,t)$ when $x\in [s_l(t),s_r(t)]$ and $E_t(\varphi )(x,t)=0$ otherwise. If $s_l(t)\in \mathscr {S}_{lT}$, $s_r(t)\in \mathscr {S}_{rT}$ and $\varphi (x,t)\in C(D_T)$, we can verify that the representation formulas

$$\begin{aligned} u(t)=e^{-\int _0^t[\gamma E_s(v)(s)+f]\textrm{d}s}E_0(u)+\delta \beta \int _0^t e^{-\int _s^t[\gamma E_\tau (v)(\tau )+f]\textrm{d}\tau } E_s(w)(s)\textrm{d}s \end{aligned}$$

and

$$\begin{aligned} v(t)=e^{-ht}E_0(v)+f \int _0^t e^{-h(t-s)} E_s(u)(s)\textrm{d}s \end{aligned}$$

hold. It follows that $u$ and $v$ can be represented by

$$u(x,t):=G(t, w(x,t))\ \text{ and } \ v(x,t):=H(t, w(x,t))$$

for $$(x,t)\in D_T.$$

Next we make a change of variable to straighten the free boundaries as in the reference^33^. Let

$$y=\frac{2s_0x}{s_r(t)-s_l(t)}-\frac{s_0(s_r(t)+s_l(t))}{s_r(t)-s_l(t)},\ z(y,t)=w(x,t).$$

Then we have

$$\begin{aligned} & \frac{\partial y}{\partial x}=\frac{2s_0}{s_r(t)-s_l(t)}:=\sqrt{\frac{B(s_l(t), s_r(t))}{p}},\ \ \ \ \ \ \ \ \ \ \frac{\partial ^{2} y}{\partial x^{2}}=0, \\ & \frac{\partial y}{\partial t}=-y\frac{s_r'(t)-s_l'(t)}{s_r(t)-s_l(t)}-s_0\frac{s_r'(t)+s_l'(t)}{s_r(t)-s_l(t)}:=-A(y, s_l(t), s_r(t)). \end{aligned}$$

And the problem (2) becomes

5 $$\begin{aligned} \left\{ \begin{array}{lll} z_t=Az_{y}+\alpha H(t, z(y,t))-\beta z+Bz_{yy},\; & -s_0<y<s_0, \ t>0, \\ s_l'(t)= \displaystyle -\frac{2\mu s_0 }{s_r(t)-s_l(t)}z_y,\quad & y=-s_0, \ t>0, \\ s_r'(t)= \displaystyle -\frac{2\mu s_0}{s_r(t)-s_l(t)}z_y,\quad & y=s_0, \ t>0, \\ z(y, t)=0,\; & y=\pm s_0, \ t>0, \\ s_l(0)=-s_0,s_r(0)=s_0,\\ z(y, 0)=(z)_0:=w_0(y),\; & -s_0\le y\le s_0, \end{array} \right. \end{aligned}$$

where

$$A=A(y,s_l(t),s_r(t))=y\frac{s_r'(t)-s_l'(t)}{s_r(t)-s_l(t)}+s_0\frac{s_r'(t)+s_l'(t)}{s_r(t)-s_l(t)},$$

$$B=B(s_l(t),s_r(t))=\frac{4s^2_0p}{(s_r(t)-s_l(t))^2}.$$

By the variation above, we can see that the free boundary fronts $x=s_l(t)$ and $x=s_r(t)$ are changed to the fixed lines $y=-s_0$ and $y=s_0$ respectively. However, the equations become more complex, as the coefficients in the top three equations of (5) now contain the unknown functions $s_l(t)$ and $s_r(t)$.

Let $k_1=-\mu w'_0(-s_0)$ and $k_2=-\mu w'_0(s_0)$ and define $\Delta _T=[-s_0, s_0]\times [0,T],$

$$\begin{aligned} \mathscr {D}_{1T}= & \left\{ z\in C(\Delta _T): \, z(y, 0)=w_0(y),\ z(\pm s_0, t)=0, \Vert z-w_0\Vert _{C(\Delta _T)}\le 1\right\} ,\\ \mathscr {D}_{2T}= & \left\{ s_l\in C^1([0,T]): \, s_l(0)=-s_0, \, s_l'(0)=k_1, \, \Vert s_l'(t)-k_1\Vert \le 1\right\} ,\\ \mathscr {D}_{3T}= & \left\{ s_r\in C^1([0,T]): \, s_r(0)=s_0, \, s_r'(0)=k_2, \, \Vert s_r'(t)-k_2\Vert \le 1\right\} . \end{aligned}$$

Note that $s_{l}(0)=-s_{r}(0)$, it is easily seen that $\mathscr {D}:=\mathscr {D}_{1T}\times \mathscr {D}_{2T}\times \mathscr {D}_{3T}$ is a complete metric space with the metric

$$\mathscr {D}((z_{1}, s_{l,1},s_{r,1}), (z_{2}, s_{l,2},s_{r,2}))= \Vert z_{1}-z_{2}\Vert _{C(\Delta _T)}+\Vert s_{l,1}'-s_{l,2}'\Vert _{C([0,T])}+ \Vert s_{r,1}'-s_{r,2}'\Vert _{C([0,T])}.$$

We now define a mapping $\mathscr {F}:\mathscr {D}\rightarrow C(\Delta _T)\times [C^1([0, T])]^2$ by

$$\mathscr {F}(z(y,t);s_l(t),s_r(t))=(\tilde{z}(y,t);\tilde{s}_l(t),\tilde{s}_r(t)).$$

where $\tilde{z}(y,t)\in C^{1+\alpha , (1+\alpha )/2}(\Delta _T)$ is the unique solution of the following initial boundary value problem

6 $$\begin{aligned} \left\{ \begin{array}{lll} \tilde{z}_t=A\tilde{z}_{y}+B\tilde{z}_{yy}+\alpha H(t, z(y,t))-\beta z,\; & -s_0<y<s_0, \ t>0, \\ \tilde{z}(y, t)=0,\; & y=\pm s_0, \ t>0, \\ \tilde{z}(y, 0)=(z)_0:=w_0(y),\; & -s_0\le y\le s_0, \end{array} \right. \end{aligned}$$

and

7 $$\begin{aligned} \tilde{s}_l(t)=-s_0-\int _0^t \frac{2s_0\mu }{s_r(\tau )-s_l(\tau )}\tilde{z}_{3y}(-s_0,\tau )\text {d}\tau , \end{aligned}$$

8 $$\begin{aligned} \tilde{s}_r(t)=s_0-\int _0^t \frac{2s_0\mu }{s_r(\tau )-s_l(\tau )}\tilde{z}_{3y}(s_0,\tau )\text {d}\tau . \end{aligned}$$

The rest of the proof is similar as those in the reference^16^. By using the $L^p$ estimates for parabolic equations and Sobolev’s embedding theorem^34^, Chapter 5, Part II, we can prove that $\mathscr {F}$ maps $\mathscr {D}$ into itself and $\mathscr {F}$ is a contraction mapping on $\mathscr {D}$ for $T>0$ sufficiently small. Then the contraction mapping theorem gives that $\mathscr {F}$ has a unique fixed point $(z; s_l, s_r)$ in $\mathscr {D}$. By the Schauder estimates again, we have additional regularity for $(z; s_l, s_r)$ as a solution of (6), namely, $s_l, s_r \in C^{1+\alpha /2} (0, T]$ and $z\in C^{2+\alpha , 1+\alpha /2} ([0, h_0]\times (0, T])$. In other words, $(z(y; t); s_l(t), s_r(t))$ is a unique local classical solution of the problem (5), and therefore $(u(x, t), v(x, t), w(x, t); s_l(t), s_r(t))$ is a unique local classical solution of the problem (2). $\square$

To prove that the local solution mentioned in Theorem 1 can be extended to $[0,+\infty )$, we also need the following estimations and Hopf lemma on the free boundary problem (2).

### Lemma 1

*If* $(u, v, w; s_l, s_r)$ *is a solution of the free boundary problem* (2) *satisfying* $t \in (0,T_0]$, *where* $T_0 \in (0, +\infty )$. *Then there exist constants* $K_1, K_2$ *and* $K_3$ *independent of* $T_0$ *such that*

$$\begin{aligned} & 0<u(x,t)\le K_1 \ \ for \ \ s_l(t)< x< s_r(t),\ 0<t\le T_0,\\ & 0<v(x,t)\le K_2 \ \ for \ \ s_l(t)< x< s_r(t),\ 0<t\le T_0,\\ & 0<w(x,t)\le K_3 \ \ for \ \ s_l(t)< x< s_r(t),\ 0<t\le T_0. \end{aligned}$$

The proof of Lemma 1 is similar to that of^21^, Lemma 2.2, so we omit the details here.

### Lemma 2

(Hopf Lemma) *Assume that* $(u, v, w; s_l, s_r)$ *is a solution to the free boundary* *problem* (2) *for* $t \in (0,T_0]$, *where* $T_0 \in (0, +\infty )$. *Then* $w_x(s_l(t),t) >0$ *and* $w_x(s_r(t),t) <0$ *for* $t \in (0,T_0]$.

### Proof

Similar to the proof in^41^, Lemma 2.1, we first straighten the right boundary $x=s_r(t)$. Let

$$\begin{aligned} y = \frac{x}{s_r(t)}, ~V(y,t) = v(x,t)~\text{ and }~W(y,t) = w(x,t). \end{aligned}$$

According to a series of detailed calculations, we can deduce that

$$\begin{aligned} \left\{ \begin{array}{ll} W_t - \frac{p}{s_r^2(t)}\cdot W_{yy} - \frac{ys_r^{'}(t)}{s_r(t)}W_y = \alpha V - \beta W, & 0< t \le T_0,~ 0<y<1,\\[6pt] W(0,t) > 0,~W(1,t) =0, & 0 < t \le T_0,\\[6pt] W(y,0) = w_0(s_0y), & 0 \le y \le 1. \end{array} \right. \end{aligned}$$

Notice that this is an initial boundary value problem with the fixed boundary. It follows from $V,W>0$ in $(0,T_0]\times [0,1)$ and the classical Hopf boundary lemma that $W_y(1,t)<0$ for $t\in (0,T_0]$. Combining with the relation $w_x=\frac{W_y}{s_r(t)}$, we infer that $w_x(s_r(t),t) < 0$ for $0<t\le T_0$. Similarly, $w_x(s_l(t),t) > 0$ for $0<t\le T_0$. $\square$

The next lemma presents that the left free boundary for (2) is strictly monotone decreasing and the right one is increasing.

### Lemma 3

*Let* $(u, v, w; s_l, s_r)$ *be a solution to the free boundary problem* (2) *defined for* $t \in (0,T_0]$, *where* $T_0 \in (0, +\infty )$. *Then there exists a constant* $K_4$ *independent of* $T_0$ *such that*

$$0<-s'_l(t),s'_r(t)\le K_4 \ \ for \ \ 0<t\le T_0.$$

### Proof

By applying Lemma 2 and Stefen conditions, we can obtain the desired result $s'_l(t)<0$ and $s'_r(t)>0$ for $0<t\le T_0$. Subsequently, it is sufficient to show that $-s'_l(t),s'_r(t)\le K_4$ for $0<t\le T_0.$ Firstly, we show that $s'_r(t)\le K_4$. And the proof of $-s'_l(t)\le K_4$ is similar.

Let

$$\Omega ^* =\bigg \{(x,t): \ s_r(t)-\frac{1}{m}<x<s_r(t)~\text{ for }~ 0<t<T,~ \text{ where }\ m>\frac{1}{s_0}\bigg \}$$

and construct an auxiliary function

$$z(x,t)=K_3[2m(s_r(t)-x)-m^2(s_r(t)-x)^2].$$

Next we will choose suitable $M$ so that $z(x,t)$ is the upper solution of $w(x,t)$ in $\Omega ^*$. Straight calculations show that

$$z_t=2 K_3 ms_r'(t)[1-m(s_r(t)-x)]\ge 0,\quad -z_{xx}=2K_3 m^2,\quad \alpha v-\beta w \le \alpha K_2.$$

It follows that

$$\begin{aligned} z_t-pz_{xx}=2pK_3 m^2+2K_3ms'_r(t)[1-m(s_r(t)-x)]\\ \ge 2pK_3 m^2\ge \alpha K_2\ge \alpha v-\beta w,\qquad \qquad \quad \end{aligned}$$

if $m \ge \frac{\alpha K_2}{2pK_3}$. On the other hand,

$$z(s_r(t)-\frac{1}{m},t)=K_3\ge w(s_r(t)-\frac{1}{m},t), \quad z(s_r(t),t)=0=w(s_r(t),t).$$

By straight computation, we get

$$z_x=-2mK_3[1-m(s_r(t)-x)]\le -mK_3,\ \ \ x\in [s_0-\frac{1}{2m}, s_0],$$

then $z_x(x,0)\le w'_0(x), x\in [s_0-\frac{1}{2m}, s_0].$ Note that $z(s_0,0)=w_0(s_0)=0,$ then $z(x,0)\ge w_0(x), x\in [s_0-\frac{1}{2m}, s_0].$ At the same time, $z(x,0)\ge z(s_0-\frac{1}{2m},0)=\frac{3 K_3}{4}, x\in [s_0-\frac{1}{m},s_0-\frac{1}{2m}].$ So, $w_0(x)\le ||w_0||_{C^1([0,s_0])}\le \frac{3 K_3}{4}.$ We then have $z(x,0)\ge w_0(x)$ in $[s_0-\frac{1}{m}, s_0]$ if $m\ge \max \{ \frac{1}{s_0}, \frac{\alpha K_2}{2pK_3}, \frac{4||w_0||_{C^1[0,s_0]}}{3K_3}\}$. Using the comparison principle yields $w(x,t)\le z(x,t)$ in $\Omega ^*$. Noticing that $w(s_r(t),t)=z(s_r(t),t)=0$, we have

$$w_x(s_r(t),t)\ge z_x(s_r(t),t)=-2mK_3.$$

Then, recalling the free boundary condition in (2) yields

$$0<s_r'(t)\le 2\mu mK_3,\quad 0<t\le T_0,$$

where *m* is independent of $T_0$. $\square$

Since (*u*, *v*, *w*) and $s_l^{'}(t),s_r^{'}(t)$ are bounded in $(s_l(t),s_r(t))\times (0,T_0]$ by constants independent of $T_0$, the global solution is guaranteed.

### Theorem 2

*The solution of problem* (2) *exists and is unique, moreover*, *it can be extended to* $$[0, T_{max})$$, *where* $$T_{max}\le +\infty$$.

In addition, the double free boundary fronts $s_l(t)$ and $s_r(t)$ not only have the character of monotonicity described in Lemma 3, but also have another significant property which will be displayed in the following theorem.

### Theorem 3

*Assume* $(u, v, w; s_l(t), s_r(t))$ *is a solution to problem* (2) *defined for* $t\in [0, +\infty )$ *and* $x\in [s_l(t), s_r(t)]$, *then we have*

$$-2s_0<s_l(t)+s_r(t)<2s_0 \ \ \text{ for } \ \ t\in [0, +\infty ).$$

### Proof

By continuity we know $s_l(t)+s_r(t)>-2s_0$ holds for small $t>0$. At the beginning, we define

$$T:=\sup \big \{s: s_l(t)+s_r(t)>-2s_0 \text{ for } \text{ all } t\in [0,s)\big \}.$$

Then, as in the reference^35^, we claim that $T=\infty$. Otherwise, $0<T<\infty$ and

$$s_l(t)+s_r(t)>-2s_0 \text{ for } t\in [0,T),\ \ s_l(T)+s_r(T)=-2s_0.$$

Hence

9 $$\begin{aligned} s_l'(T)+s_r'(T)\le 0. \end{aligned}$$

To get a contradiction, we consider the functions $z_1(x, t):=u(x, t)-u(-x-2s_0, t),$ $z_2(x, t):=v(x, t)-v(-x-2s_0, t)$ and $z_3(x, t):=w(x, t)-w(-x-2s_0, t)$ defined in the region $\Lambda :=\{(x, t): x\in [s_l(t), -s_0], t\in [0, T]\}.$ It is easy for us to verify that the pair $(z_1, z_2, z_3)$ is well-defined for $(x, t)\in \Lambda$, and it satisfies

$$z_{1t}=\delta \beta z_3-\gamma z_1 z_2-fz_1 \ \text{ for } \ s_l(t)<x<-s_0,\, 0<t\le T,$$

$$z_{2t}=fz_1-hz_2 \ \text{ for } \ s_l(t)<x<-s_0,\, 0<t\le T,\qquad \qquad$$

$$z_{3t}=\alpha z_2-\beta z_3+pz_{3xx} \ \text{ for } \ s_l(t)<x<-s_0,\, 0<t\le T \ $$

and

$$z_1(-s_0, t)=z_2(-s_0, t)=z_3(-s_0, t)=0,$$

$$z_1(s_l(t), t)<0, \ z_2(s_l(t), t)<0, \ z_3(s_l(t), t)<0, \ \text{ for } \ 0<t<T.$$

Moreover,

$$z_3(s_l(T), T)=w(s_l(T), T)-w(-s_l(T)-2s_0, T)=w(s_l(T), T)-w(s_r(T), T)=0.$$

Applying the strong maximum principle and the Hopf lemma, we deduce that

$$z_3(x, t)<0, \text{ in } (s_l(t), -s_0)\times (0, T] \ \ \text{ and }\ \ z_{3x}(s_l(T), T)<0.$$

But

$$z_{3x}(s_l(T), T)=w_x(s_l(T), T)+w_x(s_r(T), T)=-[s_l'(T)+s_r'(T)]/\mu ,$$

which implies

$$s_l'(T)+s_r'(T)>0,$$

a contradiction to (9). Hence we have proved that

$$s_l(t)+s_r(t)>-2s_0 \ \text{ for } \text{ all } \ t>0.$$

Analogously we can prove that $s_l(t)+s_r(t)<2s_0 \text{ for } \text{ all } t>0$ by considering the functions $Z_1(x, t):=u(x, t)-u(2s_0-x, t),$ $Z_2(x, t):=v(x, t)-v(2s_0-x, t),$ and $Z_3(x, t):=w(x, t)-w(2s_0-x, t)$ over the region $\Lambda ':=[s_0, s_r(t)]\times [0, T']$ with $T':=\sup \{s: s_l(t)+s_r(t)<2s_0 \text{ for } \text{ all } t\in [0,s)\}$. Thus Theorem 3 is complete. $\square$

### Remark 1

It follows from the above proof that the double free boundary fronts $s_l(t)$ and $s_r(t)$ are both finite or infinite simultaneously.

## Long time behavior of the solution

In this section, we mainly study the long-time behavior of the solution. It follows from Lemma 3 that $x=s_l(t)$ is monotonic decreasing and $x=s_r(t)$ is monotonic increasing. Therefore, there exist $-s_{l,\infty }, s_{r,\infty } \in (0,+\infty ]$ such that $\lim \limits _{t\rightarrow +\infty } s_l(t)=s_{l,\infty }$ and $\lim \limits _{t\rightarrow +\infty } s_r(t)=s_{r,\infty }$. Since the spreading of plants depend on whether $s_{r,\infty }-s_{l,\infty }=\infty$ and $\limsup \limits _{t\rightarrow +\infty }$ $(\Vert u(\cdot , t)\Vert _{C([s_l(t),s_r(t)])}+\Vert v(\cdot , t)\Vert _{C([s_l(t),s_r(t)])}+\Vert w(\cdot , t)\Vert _{C([s_l(t),s_r(t)])})=0,$ we introduce the following definition:

### Definition 1

We say that the plant fails to expand if $s_{r,\infty }-s_{l,\infty }<\infty$ and $\lim \limits _{t\rightarrow +\infty }(\Vert u(\cdot , t)\Vert _{C([s_l(t),s_r(t)])}+\Vert v(\cdot , t)\Vert _{C([s_l(t),s_r(t)])}+\Vert w(\cdot , t)\Vert _{C([s_l(t),s_r(t)])})=0,$ while invading successfully means $s_{r,\infty }-s_{l,\infty }=\infty$ and

$$\liminf \limits _{t\rightarrow +\infty }(\Vert u(\cdot , t)\Vert _{C([s_l(t),s_r(t)])}+\Vert v(\cdot , t)\Vert _{C([s_l(t),s_r(t)])} +\Vert w(\cdot , t)\Vert _{C([s_l(t),s_r(t)])})>0.$$

Before discussing the vanishing case and the conditions of the successful invasion of the model with double free boundaries, we first consider the corresponding O.D.E system of problem (2):

10 $$\begin{aligned} \left\{ \begin{array}{lll} u'(t)=\delta \beta w-\gamma uv-fu,\ & t>0,\\ v'(t)=fu-hv,\; & t>0,\\ w'(t)=\alpha v-\beta w,\ & t>0. \end{array} \right. \end{aligned}$$

It follows from the linear approximation method and Hurwitz criterion that there exists a threshold parameter $J_0\big (:=\displaystyle \frac{\alpha \delta }{h}\big )$ such that the plant tends to extinct if $0<J_0\le 1$, while the system possesses unique positive equilibrium, which is globally stable, if $J_0> 1$.

Similarly, for the system (1), in which the seeds spread while the young and old age plants do not, we can easily obtain the conclusions in the following by referring to^36^, Section 5, Chapter 11:

1. when the threshold parameter $J^D_0(\Omega )\big (:=\displaystyle \frac{\alpha \beta \delta }{h(\beta +p\lambda _1)}\big )\ge 1,$ the system (1) has an unstable homogeneous solution $O=(0,0,0).$
2. when $J^D_0(\Omega )<1,$ $O=(0,0,0)$ is the unique stable solution of (1), that is, the plant will extinct finally.

Here $\lambda _1$ denotes the first eigenvalue of the boundary value problem

$$-\Delta \psi =\lambda \psi \ \text{ in } \Omega \ \ \text{ with } \ \ \psi =0 \ \text{ on } \partial \Omega .$$

Note that, similar to the basic reproduction number of epidemic diseases, the threshold parameters $J_0$ and $J^D_0(\Omega )$ can also be defined according to the next-generation matrix method^37,38^. More calculation details can also be referred to the reference^39^, where the vector of new seeds $\mathscr {F}$ and that of transitions between compartments $\mathscr {V}$ are respectively defined by

$$\begin{aligned} (J_0):\quad \mathscr {F} = \begin{pmatrix} \delta \beta w \\ fu\\ \alpha v \end{pmatrix} ~\text{ and }~ \mathscr {V} = \begin{pmatrix} \gamma uv+fu\\ hv\\ \beta w \end{pmatrix} \end{aligned}$$

or

$$\begin{aligned} (J^D_0):\quad \mathscr {F} = \begin{pmatrix} \delta \beta w \\ fu\\ \alpha v \end{pmatrix} ~\text{ and }~ \mathscr {V} = \begin{pmatrix} \gamma uv+fu\\ hv\\ \beta w - p\lambda _1 \end{pmatrix} . \end{aligned}$$

Unlike the O.D.E system and the corresponding problem in $\Omega$ with null Dirichlet boundary condition on $\partial \Omega$ above, the interval domain for the free boundary problem (2) is changing with *t*, so the threshold parameter we given should not be a constant, but is changing with *t*. So, we introduce the threshold parameter $J^F_0(t)$ for the free boundary problem (2) by

$$J^F_0(t):=J^D_0\big ((s_l(t),s_r(t))\big )=\displaystyle \frac{\alpha \beta \delta }{h\big [\beta +p(\frac{\pi }{s_r(t)-s_l(t)})^2\big ]},$$

then the following result is obvious, see also^40^, Lemma 2.3.

### Lemma 4

$1-J^F_0(t)$ *has the same sign as* $\lambda _0$, *where* $\lambda _0$ *is the principle eigenvalue of the problem*

$$\begin{aligned} \left\{ \begin{array}{lll} -p\varphi _{xx}=-\beta \varphi +\displaystyle \frac{\alpha \beta \delta }{h}\varphi +\lambda \varphi ,\; & x\in (s_l(t),s_r(t)),\\ \varphi (x)=0,\; & x=s_l(t)\ or\ x=s_r(t). \end{array} \right. \end{aligned}$$

In fact, here

$$\lambda _0=\beta +p\big (\frac{\pi }{s_r(t)-s_l(t)}\big )^2-\frac{\alpha \beta \delta }{h}= \big [\beta +p\big (\frac{\pi }{s_r(t)-s_l(t)}\big )^2\big ]\big (1-J^F_0(t)\big ).$$

With regard to the above defined threshold parameter, we also have

### Lemma 5

$J^F_0(t)$ *is a strictly monotone increasing function of* *t*, *that is if* $t_1<t_2$, *then* $J^F_0(t_1)<J^F_0(t_2).$ *Moreover, if* $s_r(t)\rightarrow \infty ,$ *as* $t\rightarrow \infty ,$ *then* $J^F_0(t)\rightarrow J_0$ as $t\rightarrow \infty .$

Next, we discuss the two cases of “successful invasion” and “failing invasion”. And then sufficient conditions will be given to make sure that the different results will come up.

### Failing invasion

In this part, we first present the comparison principle which will be used to construct upper solution of the problem (2) to prove that the plants fail to expand. The proof of Lemma 6 is similar as ^41^, Lemma 3.1.

#### Lemma 6

(The comparison principle) *Suppose that* $\bar{s}_l(t), \bar{s}_r(t)\in C^1([0, +\infty ))$, $\bar{u}(x, t)$, $\bar{v}(x, t)$, $\bar{w}(x, t)\in C\big ([\bar{s}_l(t), \bar{s}_r(t)]\times [0, +\infty )\big )\bigcap C^{2,1}\big ((\bar{s}_l(t), \bar{s}_r(t))\times (0, +\infty )\big )$ *and*

$$\begin{aligned} \left\{ \begin{array}{lll} \bar{u}_{t}\ge \delta \beta \bar{w}-f\bar{u}, \ & \bar{s}_l(t)<x<\bar{s}_r(t), \ t>0,\\ \bar{v}_{t}\ge f\bar{u}-h\bar{v},\ & \bar{s}_l(t)<x<\bar{s}_r(t), \ t>0,\\ \bar{w}_{t}-p\bar{w}_{xx}\ge \alpha \bar{v}-\beta \bar{w},\ & \bar{s}_l(t)<x<\bar{s}_r(t), \ t>0,\\ \bar{u}(x,t)=\bar{v}(x,t)=\bar{w}(x,t)=0, \ & x=\bar{s}_l(t)\ \text{ or }\ \bar{s}_r(t),\ t>0,\\ \bar{s}_l(0)\le -s_0, \ \bar{s}_l'(t)\le -\mu \bar{w}_x(\bar{s}_l(t), t), \ & t>0, \\ \bar{s}_r(0)\ge s_0, \ \bar{s}_r'(t)\ge -\mu \bar{w}_x(\bar{s}_r(t), t), & t>0,\\ \bar{u}(x,0)\ge u_{0}(x),\ \bar{v}(x,0)\ge v_{0}(x), \ \bar{w}(x,0)\ge w_{0}(x), & -s_0\le x\le s_0, \end{array} \right. \end{aligned}$$

*then the solution* $(u, v, w; s_l, s_r)$ *to the free boundary problem* (2) *satisfies*

$$s_l(t)\ge \bar{s}_l(t), \ \ s_r(t)\le \bar{s}_r(t),\ \ \ t\in [0,+\infty )$$

$$u(x, t)\le \bar{u}(x, t), v(x, t)\le \bar{v}(x, t), w(x, t)\le \bar{w}(x, t),\ \ \ (x, t)\in [\bar{s}_l(t), \bar{s}_r(t)]\times [0, +\infty ).$$

#### Lemma 7

*If* $$s_{r,\infty }-s_{l,\infty }<\infty$$, *then* $$\lim \limits _{t\rightarrow +\infty }(\Vert u(\cdot , t)\Vert _{C([s_l(t),s_r(t)])}+$$ $$\Vert v(\cdot , t)\Vert _{C([s_l(t),s_r(t)])}+$$ $$\Vert w(\cdot , t)\Vert _{C([s_l(t),s_r(t)])})=0.$$

#### Proof

We first prove that $\lim \limits _{t\rightarrow +\infty }\Vert w(\cdot , t)\Vert _{C([s_l(t),s_r(t)])}=0.$ Assume that

$$\limsup \limits _{t\rightarrow +\infty }\Vert w(\cdot , t)\Vert _{C([s_l(t),s_r(t)])}=\varepsilon >0$$

by contradiction. Then there exists a sequence $(x_k, t_k)$ in $(s_l(t), s_r(t))\times (0,+\infty )$ such that $w(x_k, t_k)\ge \varepsilon /2$ for all $k\in \mathbb {N}$, and $t_k \rightarrow \infty$ as $k\rightarrow \infty .$ Since that $-\infty<s_{l,\infty }<s_l(t)<x_k<s_r(t)<s_{r,\infty }<\infty ,$ we then have a subsequence of $\{x_n\}$ converges to $x_0\in (s_{l,\infty }, s_{r,\infty })$. Without loss of generality, we assume $x_k\rightarrow x_0$ as $k\rightarrow \infty$.

Define $U_k(x,t)=u(x,t+t_k)$, $V_k(x,t)=v(x,t+t_k)$ and $W_k(x,t)=w(x,t+t_k)$ for $x\in (s_l(t+t_k),s_r(t+t_k)), t\in (-t_k, \infty ).$ It follows from the parabolic regularity that $\{(U_k, V_k, W_k)\}$ has a subsequence $\{(U_{k_i}, V_{k_i}, W_{k_i})\}$ such that $(U_{k_i}, V_{k_i}, W_{k_i})\rightarrow (U, V, W)$ as $i\rightarrow \infty$ and (*U*, *V*, *W*) satisfies

$$\begin{aligned} \left\{ \begin{array}{lll} U_t=\delta \beta W-\gamma UV-fU,\; & s_{l,\infty }<x<s_{r,\infty },\ -\infty<t<+\infty ,\\ V_t=fU-hV,\; & s_{l,\infty }<x<s_{r,\infty },\ -\infty<t<+\infty ,\\ W_t-pW_{xx}=\alpha V-\beta W,\; & s_{l,\infty }<x<s_{r,\infty },\ -\infty<t<+\infty .\\ \end{array} \right. \end{aligned}$$

Note that $W(x_0, 0)\ge \varepsilon /2,$ therefore $W>0$ in $(s_{l,\infty }, s_{r,\infty })\times (-\infty ,+\infty ).$

Using the similar method to prove Hopf lemma at the point $(s_{r,\infty }, 0)$ yields that $W_x (s_{r,\infty }, 0)\le -\sigma _0$ for some $\sigma _0>0.$

On the other hand, since $-s_l(t)$ and $s_r(t)$ are increasing and bounded, it follows from Standard $L^P$ theory and Sobolev’s embedding theorem^34^ that, for any $\alpha \in (0,1),$ there exists a constant *C* depending on $\tau , s_0, ||u_0||_{C^2[-s_0,s_0]}$, $||v_0||_{C^2[-s_0,s_0]}$, $||w_0||_{C^2[-s_0,s_0]}$ and $s_{l,\infty }, s_{r,\infty }$ such that

$$||w(\cdot , t)||_{C^{1+\tau , (1+\tau )/2}([s_l(t),s_r(t)]\times [0,\infty ))}+||s_r(t)||_{C^{1+\tau /2}([0,\infty ))}\le C.$$

Now, since $||s_r(t)||_{C^{1+\tau /2}([0,\infty ))}\le C$ and $s_r(t)$ is bounded, we then have $s_r'(t)\rightarrow 0$ as $t\rightarrow \infty$, that is, $\frac{\partial w}{\partial x} (s_r(t_k), t_k)\rightarrow 0$ as $t_k\rightarrow \infty$ by the free boundary condition. Moreover, the fact $||w(\cdot , t)||_{C^{1+\tau , (1+\tau )/2}([s_l(t),s_r(t)]\times [0,\infty ))}\le C$ gives that $\frac{\partial w}{\partial x} (s_r(t_k), t_k+0)=(W_k)_x (s_r(t_k), 0)\rightarrow W_x (s_{r,\infty }, 0)$ as $k\rightarrow \infty ,$ which leads to a contradiction to the fact that $W_x (s_{r,\infty }, 0)\le -\sigma _0<0.$ Thus $\lim \limits _{t\rightarrow +\infty }||w(\cdot , t)||_{C([s_l(t),s_r(t)])}=0.$

Note that $u(x,t),v(x,t)$ satisfies

$$\begin{aligned} \frac{\partial u(x,t)}{\partial t}= & \delta \beta w(x, t)-\gamma u(x, t)v(x,t)-fu(x, t),\ \ s_l(t)<x<s_r(t),\ t>0,\\ \frac{\partial v(x,t)}{\partial t}= & f u(x, t)-hv(x, t),\ \ \qquad \qquad \qquad \qquad s_l(t)<x<s_r(t),\ t>0. \end{aligned}$$

Therefore we have $\lim \limits _{t\rightarrow +\infty }||u(\cdot , t)||_{C([s_l(t),s_r(t)])}=0$ and $\lim \limits _{t\rightarrow +\infty }||v(\cdot , t)||_{C([s_l(t),s_r(t)])}=0,$ which are the desired results. $\square$

#### Theorem 4

*If* $J_0\le 1,$ *then* $s_{r,\infty }-s_{l,\infty } <\infty$ *and*

$$\lim \limits _{t\rightarrow +\infty }(\Vert u(\cdot , t)\Vert _{C([s_l(t),s_r(t)])}+\Vert v(\cdot , t)\Vert _{C([s_l(t),s_r(t)])}+\Vert w(\cdot , t)\Vert _{C([s_l(t),s_r(t)])})=0.$$

#### Proof

It follows from Lemma 7 that we only have to prove $s_{r,\infty }-s_{l,\infty } <\infty$. Let $a=\displaystyle \alpha /h$, then by tedious but fairly straightforward calculations, we can get

$$\begin{aligned} & \quad \frac{\text {d}}{\text {d}t} \int _{s_l(t)}^{s_r(t)}[au(x,t)+av(x,t)+w(x, t)] \text {d}x\\ & =\int _{s_l(t)}^{s_r(t)}\big (au_t+av_t+w_t\big )\text {d}x+s_r'(t)[au+av+w](s_r(t),t)-s_l'(t)[au+av+w](s_l(t),t)\\ & =\int _{s_l(t)}^{s_r(t)}[a(\delta \beta w-\gamma uv-f u)+a(fu-hv)+(\alpha v-\beta w+pw_{xx})]\text {d}x\\ & =\int _{s_l(t)}^{s_r(t)}pw_{xx}\text {d}x+\int _{s_l(t)}^{s_r(t)}[(a\delta -1)\beta w-a\gamma uv]\text {d}x\\ & =-\frac{p}{\mu }(s_r'(t)-s_l'(t))+\int _{s_l(t)}^{s_r(t)}[(a\delta -1)\beta w-a\gamma uv]\text {d}x. \end{aligned}$$

Integrating from 0 to $t(>0)$ gives

$$\begin{aligned} \int _{s_l(t)}^{s_r(t)}[au+av+w](x, t)\text {d}x=\int _{-s_0}^{s_0}[au+av+w](x, 0)\text {d}x+\frac{p}{\mu }\cdot 2s_0-\frac{p}{\mu }(s_r(t)-s_l(t))\\ +\int _0^t \int _{s_l(\tau )}^{s_r(\tau )}[(a\delta -1)\beta w-a\gamma uv]\text {d}x\text {d}\tau \ge 0.\qquad \qquad \qquad \end{aligned}$$

Since $(a\delta -1)\beta w(x, t)=(J_0-1)\beta w(x, t)\le 0$ for $x\in [s_l(t),s_r(t)]$ and $t\ge 0$, we have

$$\int _0^t \int _{s_l(\tau )}^{s_r(\tau )}[(a\delta -1)\beta w-a\gamma uv]\text {d}x\text {d}\tau \le 0.$$

So

$$\frac{p}{\mu }(s_r(t)-s_l(t))\le \int _{-s_0}^{s_0}[au+av+w](x, 0)\text {d}x+\frac{2ps_0}{\mu }$$

for $t\ge 0$, which in turn gives that $s_{r,\infty }-s_{l,\infty } <\infty$. Therefore the plant fails to expand. $\square$

#### Theorem 5

*If* $J^F_0(0)<1$ *and* $||u_0(x)||_{C([-s_0,s_0])}$, $||v_0(x)||_{C([-s_0,s_0])}$, $||w_0(x)||_{C([-s_0,s_0])}$ *are sufficiently small, then* $s_{r,\infty }-s_{l,\infty } <\infty$ *and* $\lim \limits _{t\rightarrow +\infty }(\Vert u(\cdot , t)\Vert _{C([s_l(t),s_r(t)])}+\Vert v(\cdot , t)$ $\Vert _{C([s_l(t),s_r(t)])}+\Vert w(\cdot , t)\Vert _{C([s_l(t),s_r(t)])})=0.$

#### Proof

We are going to construct a suitable upper solution for (*u*, *v*, *w*). Since that $J^F_0(0)<1$, it follows from Lemma 4 that there is a $\lambda _0>0$ and $0<\varphi (x)\le 1$ in $(-s_0, s_0)$ such that

$$\begin{aligned} \left\{ \begin{array}{lll} -p\varphi _{xx}=-\beta \varphi +\displaystyle \frac{\alpha \beta \delta }{h}\varphi +\lambda _0\varphi ,\; & x\in (-s_0,s_0),\\ \varphi (x)=0,\; & x=\pm s_0. \end{array} \right. \end{aligned}$$

Therefore, there exists a sufficiently small $\tau >0$ such that

$$h\gg \tau ,\ \ f\gg \tau ,\ \ \frac{\alpha \beta \delta }{h}+\lambda _0 \ge \big [\frac{\alpha \delta \beta f}{(f-\tau )(h-\tau )}+\tau \big ](1+\tau )^{2}.$$

Similarly as in the reference^16^, we set $\sigma (t)=s_0(1+\tau -\frac{\tau }{2}e^{-\tau t}),\ \ t\ge 0$ and

$$\begin{aligned} & \bar{u}(x, t)=\frac{\delta \beta }{f-\tau }\bar{w}(x, t),\ \ \qquad \qquad -\sigma (t)\le x\le \sigma (t),\ \ t\ge 0,\\ & \bar{v}(x, t)=\frac{\delta \beta f}{(h-\tau )(f-\tau )}\bar{w}(x, t),\ \ -\sigma (t)\le x\le \sigma (t),\ \ t\ge 0,\\ & \bar{w}(x, t)=\varepsilon e^{-\tau t}\varphi \big (\frac{xs_0}{\sigma (t)}\big ),\ \quad \qquad \quad -\sigma (t)\le x\le \sigma (t),\ \ t\ge 0. \end{aligned}$$

Direct computations yield

$$\begin{aligned} & \quad \bar{u}_t-\delta \beta \bar{w}+f\bar{u}\\ & =-\frac{\varepsilon \delta \beta }{f-\tau }e^{-\tau t}\varphi '\cdot \frac{xs_0\sigma '(t)}{\sigma ^2(t)}-\tau \cdot \frac{\delta \beta }{f-\tau }\bar{w}+f\cdot \frac{\delta \beta }{f-\tau }\bar{w}-\delta \beta \bar{w} \qquad \qquad \qquad \qquad \qquad \\ & \ge \big [(f-\tau )\cdot \frac{\delta \beta }{f-\tau }-\delta \beta \big ]\bar{w}=0, \end{aligned}$$

$$\begin{aligned} & \quad \bar{v}_t-f\bar{u}+h\bar{v}\\ & =-\frac{\varepsilon \delta \beta f}{(h-\tau )(f-\tau )}e^{-\tau t}\varphi '\frac{xs_0\sigma '(t)}{\sigma ^2(t)}-\big [\frac{\tau \delta \beta f}{(h-\tau )(f-\tau )}+\frac{\delta \beta f}{f-\tau }-\frac{\delta \beta f h}{(h-\tau )(f-\tau )}\big ]\bar{w}\\ & \ge \big [-\frac{\tau \delta \beta f}{(h-\tau )(f-\tau )}-\frac{\delta \beta f}{f-\tau }+\frac{\delta \beta fh}{(h-\tau )(f-\tau )}\big ]\bar{w}=0, \end{aligned}$$

$$\begin{aligned} & \quad \bar{w}_t-p\bar{w}_{xx}-\alpha \bar{v}+\beta \bar{w}\\ & =-\varepsilon e^{-\tau t}\varphi '\cdot \frac{xs_0\sigma '(t)}{\sigma ^2(t)}+\big [\beta -\tau -\frac{\alpha \delta \beta f}{(h-\tau )(f-\tau )}+\big (\frac{s_0}{\sigma (t)}\big )^2\big (-\beta +\frac{\alpha \beta \delta }{h}+\lambda _0\big )\big ]\bar{w}\\ & \ge \big \{\beta \big [1-\big (\frac{s_0}{\sigma (t)}\big )^2\big ]+\big (\frac{s_0}{\sigma (t)}\big )^2\big (\frac{\alpha \beta \delta }{h}+\lambda _0\big )- \frac{\alpha \beta \delta f}{(f-\tau )(h-\tau )}-\tau \big \}\bar{w}\\ & \ge \big \{\beta \big [1-\frac{1}{(1+\tau /2)^2}\big ]+\frac{1}{(1+\tau )^2}\big (\frac{\alpha \beta \delta }{h}+\lambda _0\big )- \frac{\alpha \beta \delta f}{(f-\tau )(h-\tau )}-\tau \big \}\bar{w}\\ & \ge \big \{\frac{1}{(1+\tau )^2}\big (\frac{\alpha \beta \delta }{h}+\lambda _0\big )- \frac{\alpha \beta \delta f}{(f-\tau )(h-\tau )}-\tau \big \}\bar{w}\ge 0. \end{aligned}$$

In addition, we have

$$\sigma '(t)=s_0\frac{\tau ^2}{2}e^{-\tau t},$$

$$-\bar{w}_x(\sigma (t),t)=-\varepsilon e^{-\tau t}\frac{s_0}{\sigma (t)}\varphi '(s_0),$$

and

$$-\bar{w}_x(-\sigma (t),t)=-\varepsilon e^{-\tau t}\frac{s_0}{\sigma (t)}\varphi '(-s_0).$$

Noticing that $\varphi '(-s_0)=-\varphi '(s_0),$ we now choose $\varepsilon =-\frac{\tau ^2s_0(1+\tau )}{2\mu \varphi '(s_0)}$ such that

$$\begin{aligned} \left\{ \begin{array}{lll} \bar{u}_{t}\ge \delta \beta \bar{w}-f\bar{u}, \ & -\sigma (t)<x<\sigma (t), \ t>0,\\ \bar{v}_{t}\ge f\bar{u}-h\bar{v},\ & -\sigma (t)<x<\sigma (t), \ t>0\\ \bar{w}_{t}-p\bar{w}_{xx}\ge \alpha \bar{v}-\beta \bar{w},\ & -\sigma (t)<x<\sigma (t), \ t>0,\\ \bar{u}(x,t)=\bar{v}(x,t)=\bar{w}(x,t)=0, \ & x=\pm \sigma (t),\ t>0,\\ -\sigma (0)<-s_0, \ -\sigma '(t)\le -\mu \bar{w}_x(-\sigma (t), t), \ & t>0, \\ \sigma (0)> s_0, \ \sigma '(t)\ge -\mu \bar{w}_x(\sigma (t), t), & t>0. \end{array} \right. \end{aligned}$$

If $\Vert u_0\Vert _{L^\infty }\le \varepsilon \varphi (\frac{s_0}{1+\tau /2})\frac{\delta \beta }{f-\tau }$, $\Vert v_0\Vert _{L^\infty }\le \varepsilon \varphi (\frac{s_0}{1+\tau /2})\frac{\delta \beta f}{(h-\tau )(f-\tau )}$ and $\Vert w_0\Vert _{L^\infty }\le \varepsilon \varphi (\frac{s_0}{1+\tau /2})$, then $u_{0}(x)\le \varepsilon \varphi (\frac{s_0}{1+\tau /2})\frac{\delta \beta }{f-\tau }\le \bar{u}(x, 0)=\varepsilon \varphi (\frac{x}{1+\tau /2})\frac{\delta \beta }{f-\tau }$, $v_{0}(x)\le \varepsilon \varphi (\frac{s_0}{1+\tau /2})\frac{\delta \beta f}{(h-\tau )(f-\tau )}\le \bar{v}(x, 0)$ and $w_0(x)\le \varepsilon \varphi (\frac{s_0}{1+\tau /2})\le \bar{w}(x, 0)$ for $x\in [-s_0,s_0]$. We now can apply Lemma 6 to conclude that $s_l(t)\ge -\sigma (t)$ and $s_r(t)\le \sigma (t)$ for $t>0$. It follows that $s_{r,\infty }-s_{l,\infty }<\lim \limits _{t\rightarrow +\infty }2\sigma (t)=2s_0(1+\tau )<\infty$ and $\lim \limits _{t\rightarrow +\infty }(\Vert u(\cdot , t)\Vert _{C([s_l(t),s_r(t)])}+\Vert v(\cdot , t)$ $\Vert _{C([s_l(t),s_r(t)])}+\Vert w(\cdot , t)\Vert _{C([s_l(t),s_r(t)])})=0$ by Lemma 7. $\square$

### Successful invasion

In some cases, plants would spread extremely fast, even out of control. In this section, we are going to give sufficient conditions to make sure that plant successfully invade into the new environment. Similarly, we first give the comparison principal, and the proof of it analogous to^41^, Lemma 3.5. Then, we prove that if $J^F_0(0)\ge 1,$ plant will spread by using the comparison principal.

#### Lemma 8

(Comparison Principle) *Suppose that* $\underline{s}_l(t), \underline{s}_r(t)\in C^1([0, +\infty ))$, $\underline{u}(x, t)$, $\underline{v}(x, t)$, $\underline{w}(x, t)\in C\big ([\underline{s}_l(t), \underline{s}_r(t)]\times [0, +\infty )\big )\bigcap C^{2,1}\big ((\underline{s}_l(t), \underline{s}_r(t))\times (0, +\infty )\big )$ *and*

$$\begin{aligned} \left\{ \begin{array}{lll} \underline{u}_{t}\le \delta \beta \underline{w}-\gamma \underline{u} K_2-f\underline{u}, \ & \underline{s}_l(t)<x<\underline{s}_r(t), \ t>0,\\ \underline{v}_{t}\le f\underline{u}-h\underline{v},\ & \underline{s}_l(t)<x<\underline{s}_r(t), \ t>0,\\ \underline{w}_{t}-p\underline{w}_{xx}\le \alpha \underline{v}-\beta \underline{w},\ & \underline{s}_l(t)<x<\underline{s}_r(t), \ t>0,\\ \underline{u}(x,t)=\underline{v}(x,t)=\underline{w}(x,t)=0, \ & x=\underline{s}_l(t)\ \text{ or }\ \underline{s}_r(t),\ t>0,\\ \underline{s}_l(0)\ge -s_0, \ \underline{s}_l'(t)\ge -\mu \underline{w}_x(\underline{s}_l(t), t), \ & t>0, \\ \underline{s}_r(0)\le s_0, \ \underline{s}_r'(t)\le -\mu \underline{w}_x(\underline{s}_r(t), t), & t>0,\\ \underline{u}(x,0)\le u_{0}(x),\ \underline{v}(x,0)\le v_{0}(x), \ \underline{w}(x,0)\le w_{0}(x), & -s_0\le x\le s_0, \end{array} \right. \end{aligned}$$

*where* $K_2$ *is the maximum ofv*(*x*, *t*), *which has been mentioned in Lemma* 1. *Then we could obtain*

$$s_l(t)\le \underline{s}_l(t), \ \ s_r(t)\ge \underline{s}_r(t),\ \ \ t\in [0,+\infty )$$

$$u(x, t)\ge \underline{u}(x, t), v(x, t)\ge \underline{v}(x, t), w(x, t)\ge \underline{w}(x, t),\ \ \ (x, t)\in [\underline{s}_l(t), \underline{s}_r(t)]\times [0, +\infty ).$$

#### Theorem 6

*If* $J^F_0(0)\ge 1$, *then* $s_{l,\infty }=s_{r,\infty } =\infty$ *and* $\liminf \limits _{t\rightarrow +\infty }\Vert w(\cdot , t)\Vert _{C([s_l(t),s_r(t)])}>0,$ *that is, the plant invades successfully.*

#### Proof

We first consider the case that $J^F_0(0):=J^F_0((-s_0,s_0))>1.$ In this case, we have that the eigenvalue problem

$$\begin{aligned} \left\{ \begin{array}{lll} -p\varphi _{xx}=-\beta \varphi +\displaystyle \frac{\alpha \beta \delta }{h}\varphi +\lambda _0\varphi ,\; & x\in (-s_0,s_0),\\ \varphi (x)=0,\; & x=\pm s_0 \end{array} \right. \end{aligned}$$

admits a positive solution $\varphi (x)$ with $\Vert \varphi \Vert _{L^\infty }=1,$ where $\lambda _0$ is the principle eigenvalue. It follows from Lemma 4 that $\lambda _0<0.$

We are going to construct a suitable lower solutions to problem (2) and then we define

$$\underline{u}(x, t)=\big (\frac{\delta \beta }{f}+\frac{\lambda _0h}{\alpha f}\big )\underline{w}(x, t),\ \underline{v}(x, t)=\big (\frac{\delta \beta }{h}+\frac{\lambda _0}{\alpha }\big )\underline{w}(x, t),\ \underline{w}(x, t)=\varepsilon \varphi (x)$$

for $-s_0\le x\le s_0, t\ge 0$, where $\varepsilon$ is sufficiently small.

Direct computations yield

$$\begin{aligned} & \underline{u}_t-\delta \beta \underline{w}+\gamma \underline{u}K_2+f\underline{u}=\big (\frac{\gamma K_2 \beta \delta }{f}+\frac{\gamma K_2 h\lambda _0}{\alpha f}+\frac{h \lambda _0}{\alpha }\big )\varepsilon \varphi (x)\le 0,\\ & \underline{v}_t-f\underline{u}+h\underline{v}=\big [h\big (\frac{\delta \beta }{h}+\frac{\lambda _0}{\alpha }\big )-f\big (\frac{\delta \beta }{f}+\frac{\lambda _0h}{\alpha f}\big )\big ]\varepsilon \varphi (x)=0,\\ & \underline{w}_t-p\underline{w}_{xx}-\alpha \underline{v}+\beta \underline{w}=-\varepsilon p\varphi ''-\alpha \big (\frac{\delta \beta }{h}+\frac{\lambda _0}{\alpha }\big )\varepsilon \varphi (x)+\beta \varepsilon \varphi (x)\\ & \qquad \qquad \qquad \qquad \quad =\varepsilon \varphi \big (-\beta +\frac{\alpha \delta \beta }{h}+\lambda _0\big )-\big (\frac{\alpha \delta \beta }{h}+\lambda _0\big )\varepsilon \varphi +\beta \varepsilon \varphi =0 \end{aligned}$$

for all $t>0$ and $-s_0<x<s_0.$ Noting that $\lambda _0<0$, we can choose $\varepsilon$ sufficiently small such that

$$\begin{aligned} \left\{ \begin{array}{lll} \underline{u}_{t}\le \delta \beta \underline{w}-\gamma \underline{u} K_2-f\underline{u}, \ & -s_0<x<s_0, \ t>0,\\ \underline{v}_{t}\le f\underline{u}-h\underline{v},\ & -s_0<x<s_0, \ t>0,\\ \underline{w}_{t}-p\underline{w}_{xx}\le \alpha \underline{v}-\beta \underline{w},\ & -s_0<x<s_0, \ t>0,\\ \underline{u}(x,t)=\underline{v}(x,t)=\underline{w}(x,t)=0, \ & x=\pm s_0,\ t>0,\\ 0=-s'_0\ge -\mu \underline{w}_x(-s_0, t), \ & t>0, \\ 0=s'_0\le -\mu \underline{w}_x(s_0, t), \ & t>0, \\ \underline{u}(x, 0)\le u_0(x),\underline{v}(x, 0)\le v_0(x),\underline{w}(x, 0)\le w_0(x), \ & -s_0\le x\le s_0. \end{array} \right. \end{aligned}$$

Hence, applying Lemma 8 yields that $u(x, t)\ge \underline{u}(x, t), v(x, t)\ge \underline{v}(x, t)$ and $w(x, t)\ge \underline{w}(x, t)$ in $[-s_0, s_0]\times [0,\infty )$. It follows that $\liminf \limits _{t\rightarrow +\infty }\Vert w(\cdot , t)\Vert _{C([s_l(t),s_r(t)])}\ge \varepsilon \varphi (0)>0$ and therefore $s_{r,\infty }-s_{l,\infty } =\infty$ by Lemma 7. $\square$

If $J^F_0(0)=1$, then for any positive time $t_0,$ we have $s_l(t_0)<-s_0$ and $s_r(t_0)>s_0$, therefore $J^F_0(t_0)>J^F_0(0)=1$ by the monotonicity in Lemma 5. Replaced the initial time 0 by the positive time $t_0$, we then have $s_{r,\infty }-s_{l,\infty } =\infty$ as above.

#### Remark 2

The above theorem implies that the plant invades successfully if there exists $t_0\ge 0$ such that $J^F_0(t_0)\ge 1$.

Theorem 5 shows that if $J^F_0(0)<1$, the plant fails to expand for small initial size and Theorem 4 implies that if $J_0\le 1$, the extinction of plant always happens for any initial values, the next result will show that spreading happens for large initial values.

#### Theorem 7

*Suppose that* $J^F_0(0)<1<J_0$, *then* $s_{r,\infty }-s_{l,\infty } =\infty$ *and* $\liminf \limits _{t\rightarrow +\infty }\Vert w(\cdot , t)\Vert$ $_{C([s_l(t),s_r(t)])}>0,$ if $||u_0||_{C([-s_0,s_0])}$, $||v_0||_{C([-s_0,s_0])}$, $||w_0||_{C([-s_0,s_0])}$ *are sufficiently large*.

#### Proof

We will construct a vector $(\underline{u}, \underline{v}, \underline{w}; \underline{s})$ such that $u\ge \underline{u}, v\ge \underline{v}, w\ge \underline{w}$ in $[-\underline{s}(t), \underline{s}(t)]\times [0,T_0]$ and $s_l(t)\le -\underline{s}(t), s_r(t)\ge \underline{s}(t)$ in $[0,T_0].$ If we can choose $T_0$ such that $J^D_0((-\underline{s}(T_0),\underline{s}(T_0)))>1,$ then $s_{r,\infty }-s_{l,\infty } =\infty$.

We first consider the following eigenvalue problem:

$$\begin{aligned} \left\{ \begin{array}{lll} -p\varphi ''-\frac{1}{2}\varphi '=\mu _0\varphi ,\; & x\in (0,1),\\ \varphi '(0)=\varphi (1)=0. \end{array} \right. \end{aligned}$$

It is well known that the principle eigenvalue of this problem is simple, and the corresponding eigenfunction $\varphi (x)$ can be chosen positive in [0, 1) and $||\varphi ||_{L^\infty }=1.$ It is easy to see that $\mu _0>\frac{1}{16p}$ and $\varphi '(x)<0$ in (0, 1]. Extending $\varphi (x)$ in [0, 1] to an even function $\phi$ in $[-1,1]$ yields that

$$\begin{aligned} \left\{ \begin{array}{lll} -p\phi ''-\frac{sgn(x)}{2}\phi '=\mu _0\phi ,\; & x\in (-1,1),\\ \phi (-1)=\phi (1)=0. \end{array} \right. \end{aligned}$$

We now construct a suitable lower solution to problem (2) and we define

$$\underline{s}(t)=\sqrt{t+\varepsilon }, t\ge 0,$$

$$\underline{u}(x, t)= \underline{v}(x, t)= e^{-lt}\int _{0}^{t}\frac{q}{(\tau +\varepsilon )^k}\phi (\frac{x}{\sqrt{\tau +\varepsilon }})\text {d}\tau ,-\sqrt{t+\varepsilon }\le x\le \sqrt{t+\varepsilon }, t\ge 0,$$

$$\underline{w}(x, t)= \frac{q}{(t+\varepsilon )^k}\phi (\frac{x}{\sqrt{t+\varepsilon }}),-\sqrt{t+\varepsilon }\le x\le \sqrt{t+\varepsilon }, t\ge 0,$$

where $\varepsilon , q, T_0, k, l$ are all positive constants and will be chosen in the following counting process.

First, using the assumption that $J_0>1$ and according to the fact that $J^D_0((-\sqrt{t},\sqrt{t}))$ $\rightarrow J_0$ as $t\rightarrow \infty$ by Lemma 5, there is $T_0>0$ satisfying $J^D_0((-\sqrt{T_0},\sqrt{T_0}))>1$.

Then, direct computation yields

$$\begin{aligned} & \underline{w}_t-p\underline{w}_{xx}-\alpha \underline{v}+\beta \underline{w}=-\frac{kq}{(t+\varepsilon )^{k+1}}\phi -\frac{qx}{2(t+\varepsilon )^{k+3/2}}\phi '-p\frac{q}{(t+\varepsilon )^{k+1}}\phi ''\\ & \qquad \qquad \qquad \qquad \qquad + \beta \frac{q}{(t+\varepsilon )^{k}}\phi -\alpha e^{-lt}\int _{0}^{t}\frac{q}{(\tau +\varepsilon )^k}\phi (\frac{x}{\sqrt{\tau +\varepsilon }})\text {d}\tau \\ & \qquad \qquad \qquad \qquad \qquad \le -\frac{q}{(t+\varepsilon )^{k+1}}\big [p\phi ''+\frac{x}{2\sqrt{t+\varepsilon }}\phi '+(k-\beta (t+\varepsilon ))\phi \big ]\\ & \qquad \qquad \qquad \qquad \qquad \le -\frac{q}{(t+\varepsilon )^{k+1}}\big [p\phi ''+\frac{sgn(x)}{2}\phi '+\mu _0 \phi \big ]=0,\\ & \underline{s}'(t)+\mu \underline{w}_x(\sqrt{t+\varepsilon },t)=\frac{1}{2\sqrt{t+\varepsilon }}+\frac{\mu q}{(t+\varepsilon )^{k+1/2}}\phi '(1)<0 \end{aligned}$$

for all $0<t\le T_0$ and $-\underline{s}(t)<x<\underline{s}(t),$ when $\varepsilon \le 1,$ $k\ge \mu _0+\beta (T_0+1)$ and $q> \frac{(T_0+1)^k}{-2\mu \phi '(1)}.$ So we have

$$\frac{q}{(t+\varepsilon )^k}\phi (\frac{x}{\sqrt{t+\varepsilon }})\le \frac{q}{(t+\varepsilon )^k}||\phi ||_{L^\infty }\le \frac{q}{(t+\varepsilon )^k}\le \frac{q}{\varepsilon ^k}.$$

It is obvious that

$$\int _{0}^{t}\frac{q}{(\tau +\varepsilon )^k}\phi (\frac{x}{\sqrt{\tau +\varepsilon }})\text {d}\tau \ge \eta$$

for $t>0$, where $\eta$ is a small positive constant.

Next, in order to get the following two inequalities in $(0,T_0]\times (-\underline{s}(t), \underline{s}(t))$,

$$\begin{aligned} & \underline{u}_t-\delta \beta \underline{w}+\gamma \underline{u}K_2+f\underline{u}=e^{-lt}\frac{q}{(t+\varepsilon )^{k}}\phi -le^{-lt}\int _{0}^{t}\frac{q}{(\tau +\varepsilon )^k}\phi \text {d}\tau -\delta \beta \frac{q}{(t+\varepsilon )^{k}}\phi \\ & \qquad \qquad \qquad \qquad \qquad +\gamma K_2 e^{-lt}\int _{0}^{t}\frac{q}{(\tau +\varepsilon )^k}\phi \text {d}\tau +fe^{-lt}\int _{0}^{t}\frac{q}{(\tau +\varepsilon )^k}\phi \text {d}\tau \\ & \qquad \qquad \qquad \qquad \qquad \le e^{-lt}\frac{q}{(t+\varepsilon )^{k}}\phi +(f+\gamma K_2-l)e^{-lt}\int _{0}^{t}\frac{q}{(\tau +\varepsilon )^k}\phi \text {d}\tau \\ & \qquad \qquad \qquad \qquad \qquad \le e^{-lt}\big [\frac{q}{\varepsilon ^{k}}+(f+\gamma K_2)T _0\frac{q}{\varepsilon ^{k}}-l\eta \big ]\le 0,\\ & \underline{v}_t-f\underline{u}+h\underline{v}=e^{-lt}\frac{q}{(t+\varepsilon )^{k}}\phi -le^{-lt}\int _{0}^{t}\frac{q}{(\tau +\varepsilon )^k}\phi \text {d}\tau + (h-f)e^{-lt}\int _{0}^{t}\frac{q}{(\tau +\varepsilon )^k}\phi \text {d}\tau \\ & \qquad \qquad \qquad \qquad \qquad \le (-l\eta +hT_0\frac{q}{\varepsilon ^k}+\frac{q}{\varepsilon ^k})e^{-lt}\le 0, \end{aligned}$$

we choose $\varepsilon \ge \varepsilon _0$, where $\varepsilon _0>0$ is sufficiently small, and $l\ge \max \{[1+(f+\gamma K_2)T_0] \frac{q}{\eta \varepsilon _0^k}, (hT_0+1)\frac{q}{\eta \varepsilon _0^k}\}$.

Finally, if $\varepsilon \le s^2_0,$ we have

$$\begin{aligned} \left\{ \begin{array}{lll} \underline{u}_{t}\le \delta \beta \underline{w}-\gamma \underline{u} K_2-f\underline{u}, \ & -\underline{s}<x<\underline{s}, \ 0<t\le T_0,\\ \underline{v}_{t}\le f\underline{u}-h\underline{v},\ & -\underline{s}<x<\underline{s}, \ 0<t\le T_0,\\ \underline{w}_{t}-p\underline{w}_{xx}\le \alpha \underline{v}-\beta \underline{w},\ & -\underline{s}<x<\underline{s}, \ 0<t\le T_0,\\ \underline{u}(x,t)=\underline{v}(x,t)=\underline{w}(x,t)=0, \ & x=\pm \underline{s},\ 0<t\le T_0,\\ -\underline{s}_0=-\sqrt{\varepsilon }\ge -s_0,-\underline{s}'(t)>-\mu \underline{w}_x(-\sqrt{t+\varepsilon }, t), \ & 0<t\le T_0, \\ \underline{s}_0=\sqrt{\varepsilon }\le s_0,\underline{s}'(t)<-\mu \underline{w}_x(\sqrt{t+\varepsilon }, t), \ & 0<t\le T_0. \end{array} \right. \end{aligned}$$

To sum up, for the sake of constructing a suitable lower solution the parameters $\varepsilon , q, T_0, k, l$ are chosen as follows

$$J^D_0((-\sqrt{T_0},\sqrt{T_0}))>1,\ \varepsilon _0\le \varepsilon \le \min \{1,s_0^2\},$$

$$k\ge \mu _0+\beta (T_0+1), \ \ \ q> \frac{(T_0+1)^k}{-2\mu \phi '(1)},$$

$$l\ge \max \Big \{[1+(f+\gamma K_2)T_0] \frac{q}{\eta \varepsilon _0^k}, (hT_0+1)\frac{q}{\eta \varepsilon _0^k}\Big \}.$$

If $\underline{w}(x,0)=\frac{q}{\varepsilon ^k}\phi (\frac{x}{\sqrt{\varepsilon }})<w_0(x)$ in $[0, \sqrt{\varepsilon }],$ plus $\underline{u}(x, 0)=0<u_0(x), \underline{v}(x, 0)=0<v_0(x)$, we can obtain that $s_l(t)\le -\underline{s}(t), s_r(t)\ge \underline{s}(t)$ in $[0,T_0]$ by using Lemma 3.2. In particular, $s_r(t)-s_l(t)\ge 2\sqrt{T_0+\varepsilon }\ge 2\sqrt{T_0}$. Note that $J^F_0(T_0):=J^D_0((s_l(T_0),s_r(T_0)))\ge J^D_0((-\underline{s}(T_0),\underline{s}(T_0)))\ge J^D_0((-\sqrt{T_0},\sqrt{T_0}))>1$, we then have $s_{r,\infty }-s_{l,\infty }=\infty$ by Theorem 7. $\square$

## Numerical simulations and discussion

In this section, we will give some numerical examples to illustrate our findings on the spreading and vanishing of the plant. Because the boundary is unknown, presenting the numerical solution is more challenging compared to problems with fixed boundaries. To achieve this, we need to estimate the positions of $x = s_l(t)$ and $x = s_r(t)$ at time *t* based on the double free boundary conditions. Subsequently, we will calculate the densities of $u,v$ and $w$ at time *t* using the Newton-Raphson method.

First, let us fix some coefficients and functions as follows:

$$\begin{aligned} \begin{array}{ll} & \beta = 0.1,~\delta = 0.01,~\gamma = 0.05,~f = 0.1,~ h = 0.03,~ s_0 = 1,\\[6pt] & u_0 = \cos (\frac{\pi x}{2}),~v_0 = 2\cos (\frac{\pi x}{2})~\text{ and }~ w_0 = 10\cos (\frac{\pi x}{2}). \end{array} \end{aligned}$$

Then, the asymptotic behaviors of the solution to problem 2 are shown by choosing different seed-diffusion rates *p*, seed production rates $\alpha$ and expanding capability $\mu$.

### Example 1

**Fig. 1:**
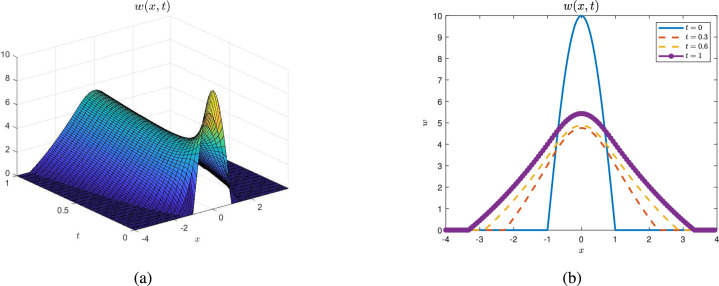
$p = 5$, $\alpha = 10$, $\mu = 1$ and $J_0^F(t) > 1$. Figures (**a**) shows that the seed population stabilizes to a positive steady stable solution when $J_0^F(t) > 1$ while pictures (**b**) is the corresponding sectional view of the seed. Figures (**a**,**b**) all implies the moving tendency of the left and right boundaries.

Fix small diffusion coefficients $p = 5$ and large expanding capability $\mu = 1$, and choose $\alpha = 10$. It is easy to see from Figure 1 that the free boundaries increase fast and the solution $w$ stabilizes to a positive equilibrium.

### Example 2

**Fig. 2:**
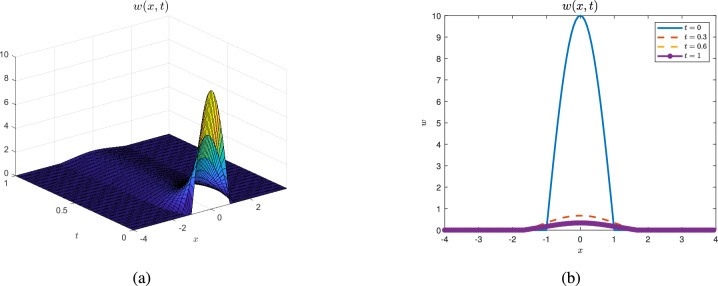
$p = 10$, $\alpha = 2$, $\mu = 0.5$ and $J_0^F(t) < 1$. Figures (**a**) shows that the seed population tends to zero when $J_0^F(t) < 1$ while pictures (**b**) is the corresponding sectional view of the seed. Figures (**a,b**) all implies the moving tendency of the left and right boundaries.

Fix large diffusion coefficients $p = 10$ and small $\mu = 0.5$, and choose $\alpha = 2$. As shown in Figure 2, the free boundaries increase slower than that in Figure 1. Moreover, the solution *w* decays to 0 quickly.

This paper focuses on an Age-Structured Continuous-Space model with double free boundaries, which describes the dynamic behaviors of invasive plants. In addition to establishing the existence and uniqueness of the solution and analyzing the properties of free boundaries for system (2), we also consider the conditions under which the plants either fail to expand or successfully invade the new environment.

We first introduce the threshold parameter

$$J^F_0(t):=\displaystyle \frac{\alpha \beta \delta }{h\big [\beta +p(\frac{\pi }{s_r(t)-s_l(t)})^2\big ]}$$

of the free boundary problem (2). This parameter depends only on the survival space $s_r(t)-s_l(t)$ when the coefficients $\alpha , \beta , \delta , h$ and diffusion coefficient $p$ are fixed. Analogous to the basic reproduction number in epidemiology^21^, $J^F_0(t)$ can be interpreted as a spatially explicit basic reproduction number. It quantifies the average number of juveniles that each adult plant can successfully establish during its life cycle under the influence of diffusion and boundary dynamics.

Based on $J^F_0(t)$, we conclude that the fate of invasive plants (whether they become extinct or expand) depends on several intertwined factors, with initial conditions playing a critical role. Specifically, when $J_0\le 1$, extinction is inevitable regardless of the initial values (Theorem 4, Fig. 2), where the threshold parameter $J_0$ was introduced at the beginning of “Long time behavior of the solution”. If $J^F_0(t_0)\ge 1$ for some $t_0\ge 0$, the plants will successfully establish themselves in a new place (Theorem 6, Remark 2 and Fig. 1). In the case where $J^F_0(0)< 1$, small initial populations lead to failure in expansion (Theorem 5), whereas large initial populations can result in successful invasion when $J^F_0(0)<1<J_0$ (Theorem 7).

It follows from the definition of $J^F_0(t)$ that the threshold exhibits a positive correlation with habitat size when other parameters are fixed. Specifically, the larger the habitat size, the greater the value of $J^F_0(t)$. Note that $J^F_0(t)\rightarrow J_0(t\rightarrow \infty )$. Consequently, if $J_0\le 1$, then $J^F_0(t)$ cannot reach 1 for any time $t > 0$. This implies that the plant’s survival area will be confined to a specific range and cannot spread indefinitely. In this scenario, “invasive plants” are unable to expand their territory and will eventually die out. Conversely, if $J^F_0(t_0) \ge 1$ for some $t_0 \ge 0$, then the plant will continue to spread and dominate in the new environment after time $t_0$(i.e., the plant invasion is successful), provided that the plant’s diffusion boundary expansion capacity $\mu$ is sufficiently large to break through the aforementioned range limit at some point in time. Finally, we consider the scenario where $J_0^F(0)<1<J_0$. If the initial values $(u_0,v_0,w_0)$ are sufficiently large, the plant expansion will likely surpass the limit and successfully spread; Conversely, if $(u_0,v_0,w_0)$ are too small, the plant will be extinct before its spatial distribution exceeds a critical limit, thereby failing to invade.

From the above theoretical analysis, it is evident that a large initial population size, extensive initial habitat area and strong boundary expansion capability significantly facilitate the successful invasion of plants into new environments. Therefore, based on these research findings, the effective prevention and control strategies for harmful invasive plants should focus on limiting their population size and habitat range. Two specific recommendations are proposed: (1) conduct comprehensive surveys to promptly identify and respond to the presence of harmful invasive plants, thereby preventing uncontrolled spread; (2) during management efforts, divide the invaded area into smaller patches, prioritizing treatment of the boundaries between these patches to isolate them, reduce the available habitat for invasive plants in each patch, and ultimately lead to their natural decline due to reduced living space.

Although the ASCS plant invasion model with double free boundaries effectively simulates plant growth and expansion, and our research findings provide valuable guidance for the prevention and control of invasive plants, it is important to recognize that plants do not necessarily diffuse uniformly in real-world scenarios. Factors such as climate and geology can cause biases in plant expansion. Therefore, we can also modify the proposed ASCS plant invasion model, and further discuss the effects of advection, nonlocal interactions, and spatial heterogeneity on plant expansion. In addition, we also suggest developing a stochastic model to investigate seed dynamics from the uncertainty model point of view.
